# From Bipotent Neuromesodermal Progenitors to Neural-Mesodermal Interactions during Embryonic Development

**DOI:** 10.3390/ijms22179141

**Published:** 2021-08-24

**Authors:** Nitza Kahane, Chaya Kalcheim

**Affiliations:** Department of Medical Neurobiology, Institute of Medical Research Israel-Canada (IMRIC) and the Edmond and Lily Safra Center for Brain Sciences (ELSC), Hebrew University of Jerusalem-Hadassah Medical School, P.O. Box 12272, Jerusalem 9112102, Israel; nitzakahane@gmail.com

**Keywords:** cell differentiation, dermomyotome, floor plate, lateral plate mesoderm, motoneurons, muscle, neural tube, notochord, sclerotome, somite

## Abstract

To ensure the formation of a properly patterned embryo, multiple processes must operate harmoniously at sequential phases of development. This is implemented by mutual interactions between cells and tissues that together regulate the segregation and specification of cells, their growth and morphogenesis. The formation of the spinal cord and paraxial mesoderm derivatives exquisitely illustrate these processes. Following early gastrulation, while the vertebrate body elongates, a population of bipotent neuromesodermal progenitors resident in the posterior region of the embryo generate both neural and mesodermal lineages. At later stages, the somitic mesoderm regulates aspects of neural patterning and differentiation of both central and peripheral neural progenitors. Reciprocally, neural precursors influence the paraxial mesoderm to regulate somite-derived myogenesis and additional processes by distinct mechanisms. Central to this crosstalk is the activity of the axial notochord, which, via sonic hedgehog signaling, plays pivotal roles in neural, skeletal muscle and cartilage ontogeny. Here, we discuss the cellular and molecular basis underlying this complex developmental plan, with a focus on the logic of sonic hedgehog activities in the coordination of the neural-mesodermal axis.

## 1. Introduction

Mesoderm formation is a multistage process. Mesoderm induction begins during gastrulation and leads to formation of the rostralmost somites. Subsequently, mesoderm induction continues after gastrulation in a special cell population termed neuromesodermal progenitors (NMPs). NMPs are located in a caudal embryonic structure, the tailbud, and represent a common pool of bipotent progenitors able to generate caudal spinal cord neurectoderm and paraxial mesoderm tissues [1,2]. As such, they are identified by the coexpression of transcription factors that characterize gastrulation, mesoderm, and neural development such as T(Brachyury), Sox2 and Nkx1/2 [3,4]. Hence, the reciprocal interactions between separate neural and mesodermal derivatives that portray patterning and morphogenetic phases of later development essentially begin early in life in the form of a common lineage.

Derivatives of NMPs comprise the paraxial mesoderm and the neural tube (NT) at spinal cord levels. Notably, human pluripotent stem cells induced in vitro to generate trunk-like NC progenitors revealed a transient intermediate neural crest (NC)/NMP state expressing both early NC (Msx1/2, Pax3, Zic1/3) and NMP markers (Bra, Sox2, Msgn1) [5,6,7]. More recently, using a trunk-specific *foxd3* enhancer to trace the lineage of NC cells in vivo, tailbud NMPs were also labeled. Subsequently, labeling was encountered in cells that express neural plate border and early NC genes and in neuronal trunk derivatives such as dorsal root ganglia [8]. Together, these results suggest that at least some trunk-level NC progenitors also derive from NMPs.

Here, we briefly and separately introduce the three interacting players in trunk patterning and morphogenesis: the paraxial mesoderm, the NT and the NC. In the forthcoming sections, we elaborate on the molecular nature of interactions between these components and on their developmental outcomes. 

### 1.1. Paraxial Mesoderm

Mesenchymal cells of the nascent paraxial mesoderm (the segmental plate) undergo a process of mesenchymal-to-epithelial transition and segment into metameric units, the somites. Recently formed epithelial somites are already heterogeneous structures, differing in properties between medial vs. lateral, and rostral vs. caudal domains [9,10,11]. These differences are determined, respectively, by graded mediolateral BMP signaling and by the dynamic rostrocaudal properties imparted by the segmentation clock [12,13]. In addition, dorsoventral differences in the somite, conveyed by notochord (No)-derived sonic hedgehog (Shh) signaling ventrally and by ectoderm-derived Wnt signaling dorsally, determine the segregation into ventral sclerotome and dorsal dermomyotome lineages, respectively, a process that becomes fully apparent upon epithelial-to-mesenchymal transition (EMT) of the former. Sclerotomal cells undergo further morphogenetic changes and form the vertebrae and ribs [10,14]. Dermomyotome precursors segregate into a variety of derivatives that include epaxial muscles of the back, hypaxial muscles of the body wall and limbs [11,15,16,17,18,19], a set of mitotic myoblasts [20], part of which generate differentiated muscle while others remain as Pax7-positive satellite cells [21,22,23], dorsal dermis [18,24], scapula blade at flank levels of the axis [25] and endothelial cells (reviewed in [11]. 

In addition to the well-established myogenic capacity of all domains of the dermomyotome (four lips and central sheet), an earlier myogenic domain comprising the medial epithelial somite was recognized years ago. It consists of a specialized group of early post-mitotic progenitors which in avian embryos, express both *MyoD* and *Myf5*. During the process of somite dissociation, these cells bend underneath the forming dermomyotome and upon delamination and slit-robo-dependent migration in the caudal to rostral direction, they differentiate into the first segmental myofibers. Together with the later contributions from the dermomyotome, they constitute the primary postmitotic myotome composed of unit-length fibers [[26,27,28,29] and reviewed in [30]].

### 1.2. Neural Tube

The NT generates the brain and spinal cord components of the central nervous system (CNS). Initially, it has a relatively simple architecture, arising when the neural plate folds to form a tube at rostral regions of the axis (primary neurulation) [31], or by secondary neurulation, that involves a mesenchymal-to- epithelial transition of NMPs to form a cord-like structure at more caudal regions of the axis. Cavitation of this cord then generates an epithelial NT [32,33], a process partially dependent on TGFβ and yes-associated protein (YAP) signaling [34]. 

In both cases, the resulting NT consists of a pseudostratified epithelium, which is only one cell thick at the earliest stages. Its dividing cells are the progenitors for all of neurons and glia. At spinal cord levels, differentiating progenitors, that completed mitosis at the ventricular (apical) domain of the neuroepithelium, migrate basally yielding a laminar structure that contains postmitotic neuronal cell bodies (mantle layer); these send their processes towards an outer marginal layer [35,36,37,38].

The spinal cord also exhibits a remarkable organization along its dorsoventral extent. Neural populations are specified by inductive signaling from opposing poles, the floor plate and the dorsal NT which later becomes the roof plate. The ventral floor plate is induced by signals from the underlying No [39], a transient mesodermal structure that later becomes the nucleus pulposus within the intervertebral disk [40]. No and floor plate produce and release Shh that acts in a graded spatial and temporal fashion to induce different cell types in the ventral spinal cord [41,42,43]. These comprise at least five progenitor (p) domains, p0–p3 and the pMN, which give rise to five distinct cell types, the ventral (V) IN, V0–V3 and motoneurons (MNs) [44,45,46,47]. The dorsal spinal cord contains at least six dorsal progenitor (dP) domains, dP1–dP6, that differentiate into the dorsal interneuron populations 1–6 [36]. These are patterned by BMP and Wnt morphogens initially derived from the dorsal NT and later from the roof plate itself which likely affects subsequent aspects of interneuron differentiation [48,49,50,51,52]. Notably, retinoic acid (RA) from the adjacent paraxial mesoderm was also shown to influence diverse aspects of NT development along the entire dorsoventral extent of the neuroepithelium [47,53,54]. 

Under the influence of these morphogens, distinct domains of progenitors are generated that express a unique combination of transcription factors specifying their identities. Then, a cross-inhibitory talk between cells expressing the above factors further refines the pattern and leads to formation of discrete populations of neurons, as described above. This combinatorial network of transcription factors not only regulates progenitor cell position and patterning, but also further differentiation and later connectivity and function [55]. 

### 1.3. The Neural Crest

The development of the dorsal NT is of particular interest as in addition to generating specific components of the CNS, it is also the source of NC progenitors that yield most of the peripheral nervous system as well as of additional non-neural derivatives such as melanocytes, ectomesenchyme and endocrine cells [56]. In fact, three cell populations sequentially form in the dorsal NT. First, NC cells that actively proliferate, undergo EMT, leave the NT and engage in migration as mesenchymal cells [56,57]. Second, roof plate cells, which exit the cell cycle, regain epitheliality and baso-apical polarity [58,59]. Third, spinal interneurons whose specification and/or differentiation are induced by the roof plate that acts as an organizing center (Section 1.2) [60].

Growing evidence substantiates the significance of interactions between the above NMP derivatives; from NT patterning and differentiation of specific cell types to induction of NC cells, control of NC emigration and migration followed by peripheral nervous system segmentation, all of which are modulated by mesodermal signals. Reciprocally, survival and proliferation of paraxial mesoderm, control of myogenesis and chondrogenesis are elicited by NT, NC and No-derived factors. The molecular basis of these processes is discussed in the next sections.

## 2. Factors Underlying the Choice of NMPs to Generate Neural or Mesodermal Fates

NMPs are Sox2+/Brachyury+ cells that form the bulk of neural and paraxial mesoderm progenitors of the posterior trunk region [2]. Whether NMPs constitute a population of bipotent cells or alternatively, a mixture of precursors separately committed to each lineage remained unclear. Recent lineage tracing in the chick embryo using a barcoded retroviral library and the Brainbow method for in vivo clonal analysis, revealed a resident cell population, mapped to the anterior primitive streak epiblast, that contains single cells contributing to both neural and mesodermal lineages in trunk and tail, and further characterized their dynamics and molecular profile [61]. NMPs can either self-renew to maintain the bipotent state, or differentiate either as paraxial mesoderm or neuroectoderm. Most notably, the principal molecular components of the network regulating the progenitor state and its differentiation are conserved across species. They include canonical Wnt, FGF, and RA acting in combination with the transcription factors T (Brachyury), Tbx6 and Sox2. 

In zebrafish, FGF plays a dual role, it activates Brachyury in the early gastrula, but it represses both Brachyury and Sox2 at the later NMP stage. Consequently, cells commit to a mesodermal fate, a process resulting from the coordinated activities of Wnt signaling, responsible for cellular epithelial-to-mesenchymal transition (EMT), and FGF that ends the process [62]. Kinney et al. [63], showed the existence of a developmental checkpoint during mesoderm induction, ensuring that Sox2-expressing cells do not migrate into the mesoderm by maintaining a partial EMT state [64]. The underlying mechanism involves synergy between Sox2 and the mesoderm-inducing Wnt pathway. When Wnt signaling was inhibited in Sox2-expressing cells the latter entered the mesoderm while keeping a partial EMT configuration, and formed an ectopic spinal cord instead of the mesoderm [63]. This checkpoint was interpreted to be critical for preventing ectopic spinal cord formation in place of the mesoderm, thus coordinating morphogenetic movements with proper cell fate acquisition.

Consistently, loss of either Brachyury, Tbx6, Fgfr1 or Wnt3a leads both to shortened axes, and the ectopic production of neural tissue at the expense of somitic mesoderm [65,66,67,68]. Lineage-tracing studies in mouse mutants lacking Wnt3a/β-catenin signaling provided genetic evidence that trunk progenitors normally fated to enter the mesoderm can be redirected towards the neural lineage. This further substantiated the notion that Wnt3a/β-catenin signaling directs trunk progenitors towards a paraxial mesoderm fate. To note is, that in the same study, the authors additionally found that Wnt3a positively supports maintenance of the dual progenitor state [69]. Along this line, during midtrunk formation, Wnt/β-catenin signaling was shown to expand the number of Sox2+T+ NMPs and maintain the appropriate level of Brachyury in the NMP population [70]. 

In contrast, RA was shown to promote neural differentiation of NMPs [reviewed in [4]]. Mouse Raldh2-/- embryos, lacking RA synthesis, exhibited decreased Sox2+ and Sox1+ neuroectodermal progeny and increased Tbx6+ presomitic mesodermal progeny. In addition, somites were smaller in these mutants due to ectopic caudal FGF8 activity. This Raldh2-/- small somite defect was rescued by treatment with an FGF receptor antagonist, suggesting that RA activity in undifferentiated neural progenitors is sufficient to restrict caudal Fgf8 expression in both neuroectoderm and mesoderm and hence control neurogenesis as well as somite size. Thus, the inhibitory cross-talk between RA and FGF accounts for the coordination of somitogenesis with neurogenesis [71].

RA and Wnt signals also stand in an inhibitory interaction. Using single-cell transcriptomics, Gouti et al. [72], identified the molecular signature of mouse NMPs, and together with genetic perturbations, the authors uncovered a transcriptional network and feedback interactions that integrate these opposing activities to regulate the bifurcation of neural and mesodermal fates. RA, whose levels are tightly regulated in the tailbud region both by synthesis and degradation, was found to play a dual role. First, it induces NMPs. As cells leave the NMP zone they differentiate to mesoderm progenitors expressing Bra/Msgn1/Tbx6, which results in upregulation of Aldh1a2, the RA-synthesizing enzyme. In turn, increased synthesis of RA in nascent mesodermal cells located in proximity to the niche promotes Sox2 expression and the progressive differentiation of NMPs to neural progenitors [72].

## 3. Mutual Interactions between Neural and Mesodermal Progenitors Shape the Body Plan

### 3.1. Mesoderm-Neural Tube Interactions

A close spatial correlation is observed in zebrafish embryos between the repetitive pattern of motoneuron progenitor distribution in the NT and the changing architecture of the adjacent somites, suggesting that signals from paraxial mesoderm specify different motoneuron subtypes. Results of grafting experiments confirmed this notion [73,74]. Furthermore, motoneuron specification was disturbed in spadetail (spt) as well as in additional zebrafish mutants, all exhibiting defective formation of the trunk paraxial mesoderm [75,76,77,78,79]. Additionally, heat-shock-induced defects in somite segmentation altered the position of motoneurons and their axonal morphology [80,81].

Furthermore, in avian embryos, the progression of neurogenesis in the NT is subservient to the dynamics of paraxial mesoderm development. Whereas presomitic mesoderm and FGF signaling maintain expression of caudal neural genes in the prospective spinal cord, neuronal differentiation is repressed by them and induced instead by factors emanating from the segmented somites. Inhibition of FGF activity was not sufficient to promote neuronal differentiation, suggesting the need for additional somite-derived factors [82]. RA was later identified as the somitic signal that antagonizes FGF and stands in a reciprocal gradient of expression along the rostrocaudal axis. By attenuating Fgf8 activity in both neuroepithelium and paraxial mesoderm, RA controls the positioning of somite boundaries, expression of ventral neural patterning genes and neuronal differentiation, thereby coordinating a neural-mesodermal cross-talk [54]. Thus the RA/FGF antagonism is a reiterative motif during early fate choices of NMPs and later once mesoderm and neuroectoderm are established as separate entities. 

The recurring roles of the same set of factors throughout development, impose the need for analysis of neural-mesodermal interactions at specific stages and time windows, a technically challenging task in mammalian embryos. To overcome these limitations Veenvliet et al. [83] produced highly organized “trunk-like structures” comprising both NT and somites from pluripotent mouse embryonic stem cells. Upon embedding in extracellular matrix, cell aggregates initially formed, which then self-organized and segregated into these two main tissues. Comparative single-cell RNA sequencing analysis confirmed that this in vitro process resembles molecularly the progressive development of the mouse trunk. As a proof of concept, the authors implemented compound organoids in which mesodermal Tbx6 was knocked out. These structures developed additional NTs, similar to the embryo mutants, further confirming the role of mesodermal factors in neural patterning, and providing a promising platform for future research on tissue interactions. Reciprocally, somite patterning requires signals from adjacent cell types as revealed by grafting experiments in chicks. For example, the *paraxis* gene, important for maintaining somite epitheliality, is first expressed in epithelial somites and then in the dermomyotome. Paraxis is a target for signals released from ectoderm and/or NT [84]. Additional signals operating between somitic and NT tissues involve a third cell type, the NC, and will be discussed in the next section.

### 3.2. Interactions between NT and Somites Control Multiple Aspects of NC Development

#### 3.2.1. NC Induction

Formation of the NC is influenced by interactions with mesodermal tissue [see [85,86,87] for comprehensive reviews]. The dorsolateral marginal zone (DLMZ) of the Xenopus gastrula that generates paraxial and intermediate mesoderm is the source of NC-inducing signals, as revealed by recombination experiments [88,89,90]. Further maintenance of NC markers was also shown to require paraxial mesoderm [88,91]. The DLMZ expresses multiple Wnt and FGF ligands and the BMP antagonist Chordin [88,89,92,93], likely to mediate these events. Likewise, in avians, recombination between nascent neural tissue and somites or lateral mesoderm also generated NC-derived melanocytes [94]. 

#### 3.2.2. The Timing of NC EMT

A significant body of evidence, primarily stemming from avian embryos, relates the onset of NC migration in the trunk with somitogenesis and subsequent somite dissociation [95,96,97]. This suggested that the paraxial mesoderm regulates aspects of NC EMT and emigration. 

**Figure 1:**
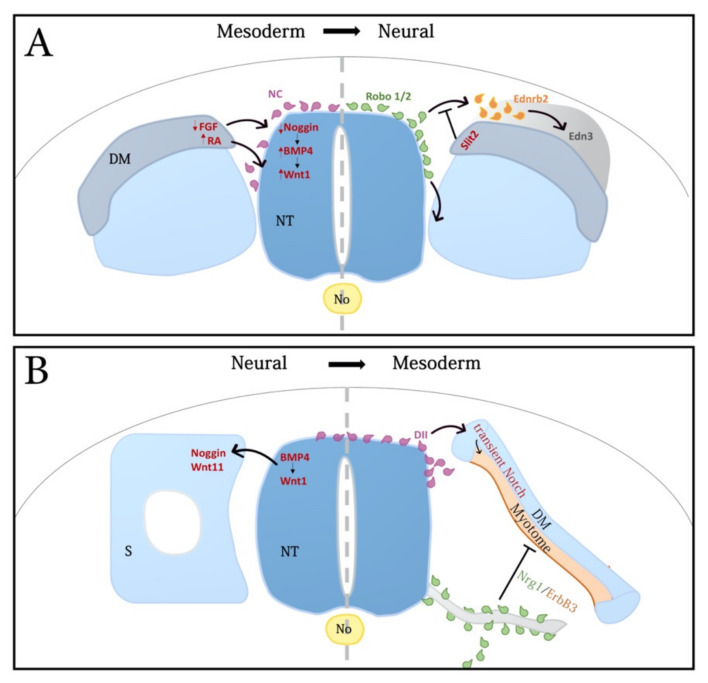
NC-mesoderm interactions. (**A**) Left side: The medial lip of the nascent dermomyotome (DM) controls the timing of NC delamination. In the early dorsal neural tube (NT), prior to the onset of NC emigration, levels of noggin are high, in part due to somite-derived FGF signaling, thereby inhibiting the activity of BMP4 and consequent NC delamination. During somite dissociation, decreasing FGF signaling results in reduced transcription of noggin in the dorsal NT. Consequently, inhibition on BMP4 is relieved, Wnt1 transcription is stimulated and NC emigration is set in motion. In parallel, increasing retinoic acid (RA) levels in the DM act upon the dorsal NT to induce Wnt1 and trigger NC cell emigration (purple cells). (**A**) Right side: The DM and later dermis influence the choice of pathways followed by NC cells. During the early stage of NC migration, DM cells express the secreted ligand Slit2, which acts upon Robo1/2-expressing NC cells (green) to prevent them from entering prematurely the dorsolateral pathway. At a later stage of migration, the Edn3 ligand, expressed in the somite-derived dermis and in the ectoderm attracts melanoblasts (yellow) that express the Ednrb2 receptor following delamination from the neural tube; this interaction guides them into the dorsolateral pathway. (**B**) Left side: The dorsal neural tube (NT) patterns the medial DM. NT-derived Bmp4 and Wnt1 signal the adjacent dorsal somite (S) to promote a medial identity, the development of the medial lip of the DM, and the expression of genes such as noggin and Wnt11, respectively. (**B**) Right side: Migrating NC cells regulate myogenesis. Migrating NC cells (purple) contact the dorsomedial lip of the DM and signal through Delta1 (Dll) to transiently activate Notch in the DM; this results in enhanced myogenesis at the expense of Pax7-positive progenitors in the epithelium. Migrating NC cells fated to become Schwann cells (green) along peripheral nerves signal through Nrg1/ErbB3 to the central and hypaxial DM and myotome (pink) to maintain the progenitor state while inhibiting myogenic differentiation. Stippled grey lines separate the NT in halves to illustrate different processes in each. See text for details and references. Abbreviations: No, notochord; Scl, sclerotome.

An interplay between noggin and BMP4 in the dorsal NT generates a graded activity of BMP that, via regulation of Wnt1 transcription and Wnt-dependent canonical signaling, triggers delamination of NC progenitors and the consequent onset of cell migration [98,99]. This rostral-to-caudal gradient of BMP4 activity is generated in spite of a constant level of BMP4 mRNA production along the dorsal NT by virtue of an opposing, decreasing gradient of noggin transcription and activity that correlates with somite development. Downregulation of noggin progressively relieves inhibition of BMP and allows NC EMT. Somitic factors were suggested to influence the levels of noggin mRNA in the NT. Consistent with this notion, grafting experiments revealed that dissociating, but not younger somites, emit an inhibitor of noggin production in the dorsal NT, thereby coupling the time of EMT with the development of the somites as suitable substrates for subsequent NC migration [100]. Opposing gradients of FGF8 and RA, apparent in the paraxial mesoderm, were reported to control the timing of NC EMT, in part through the modulation of specific aspects of BMP and Wnt signaling [101] (Figure 1A). These results further exemplify the importance of reiterative molecular modules as mediators of the dynamics of trunk development.

#### 3.2.3. Segmental Migration of NC and Patterning of the PNS

The PNS of higher vertebrates is segmented to align the peripheral ganglia and nerves with the vertebrae. This pattern is established during embryogenesis, when vertebrae develop from the somite-derived sclerotome and NC cells preferentially migrate into the rostral sclerotome halves. Grafting experiments in avian embryos showed that the metameric arrangement of the PNS depends upon the rostro-caudal alternation of sclerotomal properties [102,103,104,105], and so is the development of specific components of the vertebrae [106,107]. Several gene families were shown to mediate segmental NC migration primarily through repulsive interactions between caudal sclerotome and NC cells; these include Eph and ephrins, F-spondin, neuropilins and semaphorins, T-cadherin, etc. [108,109,110,111,112,113,114]. Whereas some of the pathways operate sequentially, others might act cooperatively as part of a regulatory network that ensures proper segmental patterning. Notably, to date, no experiments that examined the existence of such a network have been published.

#### 3.2.4. Melanoblast Migration along the Dermomyotome-Derived Dermis

The latest NC cells to exit the NT produce melanocytes and migrate along a dorsolateral pathway through the nascent dermis [115]. These NC-derived melanocytes colonize the epaxial region of the body and their patterning results from a close interaction between the NC progenitors and somite-derived dermis [116]. It is worth mentioning that early-emigrating Schwann cell progenitors are also an important source of melanocytes yet they differentiate later and are restricted to the hypaxial body domain including limbs [116,117].

The choice of ventral vs. lateral migration pathways seems also to depend on somitic factors. Dermomyotome-derived Slit2 represses the entry of Robo-expressing NC cells into the lateral pathway thus confining the migration of early NC cells to the ventral route [118] (Figure 1A). Additional repulsive cues are present in both the caudal sclerotome and the dorsolateral pathway such as ephrins, F-spondin, chondroitin sulfate proteoglycans and PNA-binding molecules [111,119,120,121]. On the other hand, positive chemotactic guidance molecules such as the Ednrb2 and EphB2 receptors [122] were identified. Ednrb2 is upregulated in melanoblasts prior to entering the dorsolateral domain, and endothelin3 (ET3), its ligand, is expressed by cells of the ectoderm, dermomyotome and the mesenchymal dermis [123,124,125,126] (Figure 1A). Of interest is that even if initiation of dorsolateral migration follows the appearance of the dorsal dermis, misexpression of Ednrb2 in NC progenitors at earlier stages is sufficient for driving cell migration prematurely between ectoderm and epithelial dermomyotome [125]. Possibly, high levels of the Ednrb2 receptor are dominant over inhibitory cues present in the superficial pathway at early stages. 

### 3.3. NC-Mesoderm Interactions in the Regulation of Myogenesis

An extensive crosstalk between NC cells and the adjacent mesoderm was recognized at all levels of the neuraxis. In the head, sequential interactions between NC and the cranial mesoderm shape craniofacial morphogenesis, development of the musculature and aspects of mutual cell differentiation [127,128,129,130,131,132,133,134,135].

In the trunk, NT/NC-somite interactions play a fundamental role in development of somite derivatives. The early dorsal NT regulates specific aspects of dermomyotome development through BMP4 activity [136], as well as subsequent formation of the dorsal dermis through neurotrophin 3 [137]. In addition, Wnt signals pattern the medial dermomyotome [138,139,140,141,142], and Wnt1 from the dorsal NT acts through the canonical pathway to control expression of Wnt11 in the medial dermomyotome, which in turn orients myocyte elongation [143] (Figure 1B).

Early migratory NC cells, transiently contacting the medial dermomyotome, provide promyogenic signals via activation of the Notch pathway [144]. Furthermore, NC-derived Neuregulin1 acting through the ErbB3 receptor, regulates murine muscle development by maintaining the Pax7-positive progenitor pool and preventing premature myogenic differentiation [145] (Figure 1B) [highlighted in [146]].

## 4. Shh, an Axial Midline Morphogen, Is Essential for Neural and Mesodermal Development

Shh is a well-studied morphogen, that plays essential roles in development of both NT and somites [44,147,148,149,150,151]. Signaling by this ligand is initiated by binding to the transmembrane receptor patched (Ptc), that represses the pathway in its absence and is also a direct transcriptional target of Shh [152,153]. Ligand binding to Ptc reveals repression on smoothened, a key effector essential for hedgehog signal transduction [154]. Transduction of Shh signaling is believed to take place in apically localized cilia where a dynamic behavior of Ptc and smoothened was documented to modulate Gli transcriptional activity [155,156,157,158,159,160]. Shh signaling is highly regulated by negative and positive modulators. In addition to Ptc1, hedgehog interacting protein (Hhip) and Gli1 are also direct targets of Shh and the former two also inhibit its activity [161,162]. Sulfatase1 [163], Boc, Gas and Cdo [164,165] enhance ligand activities and are expressed in NT and/or developing mesoderm [166].

Shh is synthesized and secreted by the No and then also the floor plate of the NT. Its functions as a morphogen are exemplified by induction of distinct ventral cell identities in the overlying NT via a mechanism that depends on relative concentrations and duration of exposure [43,167,168]. Likewise, a concentration gradient of Shh was suggested to control the in vitro induction of sclerotome and myotome [149,166]. Moreover, its activity continues beyond the patterning stage to regulate cell proliferation, survival and differentiation in both systems [[169,170], and see below]. 

### 4.1. Shh in Muscle Development

In zebrafish, different levels and durations of Shh signaling specify distinct myotomal cell types [162,171,172,173,174]. No-derived Shh is also involved in regulating mesoderm precursor cell survival, proliferation, and differentiation. In the chick, surgical ablation of the NT and No strongly affected epaxial muscle, vertebrae and rib formation due to cell death in the somites. Notably, grafting of either the ventral NT or the No, or even of cells secreting Shh in place of the deleted axial organs rescued formation of epaxial muscles, ribs and vertebrae. These results suggested that Shh emanating from the floor plate and/or the No is required for survival of both myogenic and chondrogenic cell lineages [175]. In addition, Shh regulates *Myf5* expression in primary epaxial myoblasts [176], and ectopic application of Shh causes premature myoblast differentiation at the expense of *Pax3* expression [15,176,177,178,179].

**Figure 2:**
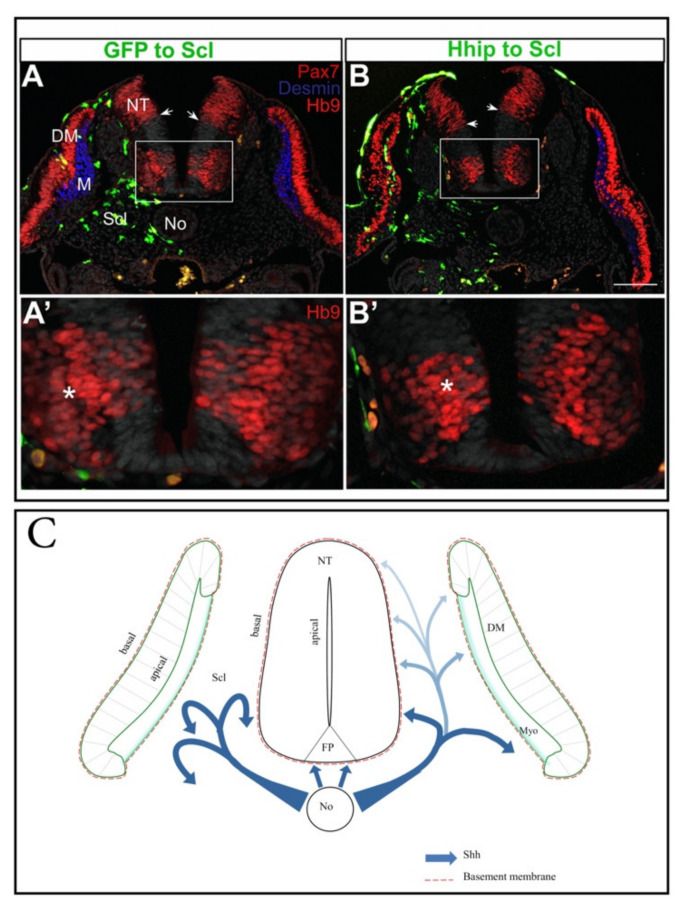
Notochord-derived Shh traversing the sclerotome affects both myotome and motoneuron differentiation. (**A**,**B**) Electroporation of control GFP (green) (**A**) or hedgehog-interacting protein (Hhip1) (green in (**B**)) to the prospective sclerotome at the flank level of the axis. A day later a reduction in myotome size (blue desmin staining) is apparent adjacent to the transfected cells. In addition, note ventral shift of the Pax7+ boundary in (**B**) (small arrows) indicative of a dorsalization of NT properties. (**A’**,**B’**) Higher magnification of the insets in (**A**,**B**), respectively, depicting a reduction of Hb9+ motoneurons in (**B**) upon Hhip1 treatment compared to the control side and to control GFP (**A’**). Asterisks (*) denote motoneurons adjacent to electroporated sclerotomes. Similar results were obtained with a membrane-tethered Hhip and with Ptc plasmids [see [188] for further details]. (**C**) A proposed model for the activity of notochord (No)-derived Shh. The No secretes Shh that acts on the ventral neural tube (NT) and also traverses the sclerotome (Scl) which is both a pathway for ligand movement and also a target of its activity. A ventral to dorsal gradient of ligand is created in Scl (blue arrows) [166], that influences both myotome as well as motoneuron development. Shh is thus presented to the target epithelial cells via its basal domain, probably by initial association with the laminin-containing basement membrane (red line). In addition, Shh traversing the sclerotome is also required for aspects of sclerotomal development (blue arrows on left side of image). Scale Bar, 50 μM.

Early muscle formation is actually subdivided into two main phases. An initial patterning phase composed of postmitotic, unit-length myofibers [16,17,18,27,30,180,181,182], and a second phase characterized by cell expansion. The latter is associated with dissociation of the central sheet of the dermomyotome that produces dermis and Pax3/7-positive myoblasts. These remain mitotically active within the myotome [20,183] and later in development generate either fibers or satellite cells, the adult muscle stem cells [21,22,184]. Using a mouse transgenic line, a ventrodorsal activity gradient of Shh/Gli signaling was directly visualized spreading from the No through the sclerotome [166]. In addition, in chick embryos, specific inhibition of Shh activity in sclerotome, impaired dermomyotome cell proliferation and epitheliality generating smaller dermomyotomes whose epithelial configuration is disrupted, primarily in their central domain (Figure 2B). Furthermore, terminal differentiation of muscle progenitors that entered the primary myotomal domain was significantly reduced with residual Pax7-positive progenitors instead of differentiated myocytes (Figure 2B) [166]. Thus, the sclerotome is an important pathway for Shh transport and distribution to target cells. Notably, somewhat later, dissociating dermomyotome progenitors become refractory to Shh signaling, showing that the activity of Shh is only temporary, thereby allowing a dynamic transition from muscle patterning to growth [166].

### 4.2. Shh and Cartilage Development

The somite-derived sclerotome is not only a pathway for Shh distribution, it is also an important target of activity. The sclerotome is a transient, embryonic tissue composed of mesenchymal cells that derives from the ventromedial region of the somite. The localization and specification of the sclerotome is a tightly controlled process stimulated by Shh signaling from the floor plate of the NT and/or No, which induce expression of early sclerotome markers such as *Pax1*, *Pax 9*, *Nkx3.2* and *Mfh1* [14,147,185,186,187]. Selective inhibition of Shh signaling in the ventral somite results a day later, in formation of smaller sclerotomes, suggesting that Shh is also involved in size control [188]. Similar to the antagonistic activities of BMP and Shh signaling in NT development, in the mesoderm, opposing functions of lateral plate mesoderm-derived BMP and midline-derived Shh allow sclerotome differentiation. To this end, the No not only expresses Shh but also noggin, a powerful BMP antagonist [189]. 

The sclerotome forms most components of the axial skeleton: vertebrae, ribs, cartilaginous end plates, the annulus fibrosus, and also tendons and ligaments via an intermediate domain, the syndetome [190,191,192]. Not surprisingly, being produced by the No, Shh was shown to be necessary for the formation of the sheath encircling the No itself. Removal of Shh signaling resulted in formation of small and dispersed nuclei pulposi, remnants of the primitive No that localize to the middle part of the intervertebral discs. Hence, Shh-dependent sheath integrity is probably responsible for maintenance of the rigid, rod-like structure that characterizes the No [193].

As mentioned above, sclerotomal precursors also generate the ribs. Loss of the proximal part of the ribs is observed in Shh null mutants [185], and in mouse mutants depleted of the Shh-dependent sclerotomal marker gene *Pax1* [194].

Although midline-derived Shh is necessary both for myotome and sclerotome formation, direct myotome-sclerotome interactions, and subsequent intercostal muscle-rib communication, were also documented as being important for proper rib growth and patterning [195,196]. These are likely to take place in a Shh-independent mode. 

In this context, it is worth mentioning that neural-skeletal connections also extend into adult life. Neurons of sympathetic, parasympathetic, and sensory branches communicate with cells of the bone microenvironment and regulate bone development, bone mass accrual, bone remodeling, and even spread of metastatic cells. Understanding the precise functions of innervation in the control of bone homeostasis throughout the organism’s life span may lead the way to new therapeutic approaches [197].

### 4.3. Shh in the Coordination of Neural-Mesodermal Development

As discussed in the previous sections, the NT and mesoderm are two developmentally and functionally interconnected systems, and Shh plays pivotal, yet time-limited, roles in their establishment. A question stemming from these findings, is whether the effects of Shh on either tissue are independent of each other or interrelated. As for neural development, does the NT receive Shh directly from the producing sources (No and/or floor plate), or given the ligand is released into the mesoderm, can the latter serve as a pathway from which Shh affects aspects of both NT and mesoderm development? Two findings obtained in avian embryos support the latter notion. First, reducing the amount of Shh uniquely in the sclerotome by hedgehog-interacting protein, by a membrane-tethered version of this inhibitor that is unable to diffuse away from transfected cells, or by missexpression of the transmembrane receptor Ptch1, significantly reduced motoneuron numbers and concomitantly reduced myotome development [166,188] (Figure 2A–B’). The observed phenotypes were a specific and direct consequence of Shh depletion. Furthermore, deletion of the floor plate did not affect either process, suggesting that, at a post-neurulation stage, the sclerotome constitutes a dynamic substrate of No-derived Shh that acts both on motoneuron as well as on myotome development. Second, grafting No fragments adjacent to the basal, sclerotomal side of the NT profoundly affected motoneuron development when compared to similar grafts applied in the NT lumen, e.g.; at the apical side. This suggested that active ligand must be presented to the epithelium from its basal side that is in contact with the sclerotome through which Shh transits and that also serves as a pathway to affect muscle and sclerotome development (Figure 2C) [188]. The significance of these findings is twofold: first, it presents a novel pathway through which No-derived Shh disperses to promote aspects of neural development; second, it suggests that by affecting both motoneuron and muscle differentiation, a single ligand coordinates initiation of future neuromuscular organization (Figure 2).

## 5. Conclusions and Future Perspectives

Investigating development of the neural-mesodermal axis enables us to recognize the logic underlying establishment of the body plan. The complex events leading from NMPs to paraxial mesoderm and NT, and ensuing interactions leading to coordinated formation of their respective derivatives, embody most of the basic processes in embryogenesis, from cell fate decisions to cell proliferation, migration, terminal differentiation and patterned morphogenesis. 

Clonal approaches in vitro and in vivo recently showed the bifated nature of NMPs [61] and additional studies begun addressing the molecular network responsible for fate segregation (see Section 2). Much is still to be uncovered regarding the relationship between cell fate acquisition and morphogenesis, by addressing dynamic processes such as EMT and cell migration. 

An intriguing question is what NC populations emanate from NMPs [8], are these primarily neural progenitors or also melanocytes? Additional labeling strategies should be implemented to address the lineage of the trunk NC back to these fascinating postgastrulation progenitors, followed by exploring mechanisms of lineage segregation. Advances in protocols for the establishment of compound organoids should complement in vivo approaches, primarily for research into mouse and human systems. These should be useful in uncovering the feedback mechanisms and transcriptional modules operating downstream of Wnt, FGF, and RA and perhaps of additional factors. Clarifying the nature of cross-inhibitory interactions at transcriptional and post-transcriptional levels will enable a better understanding of how different fates are established and further refined. 

Another captivating topic concerns the mode of transport of morphogens towards the target cells. In the context of mesoderm-neural interactions, we proposed that a significant fraction of Shh operating on the neuroepithelium stems from the No via a sclerotomal pathway that, at somitic stages, is more active than the floor plate in promoting motoneuron development [188].

How is Shh transported through the sclerotome? Possible models could involve packaging of the ligand in No-derived exosomes [198], diffusion of Shh released by metalloproteinases in a lipid-free form [199], secretion as multimeric complexes [200] and/or via carrier-mediated transport through the extracellular space [201]. The precise mechanism responsible for Shh transport in this context remains to be unraveled.

Although apical cilia act as activity centers of Shh signal transduction, our findings would suggest that neuroepithelial cells sense Shh from their basal pole that faces the sclerotome. This is consistent with enhanced motoneuron differentiation occurring upon basal but not apical presentation of a No under experimental conditions, and also in normal development as the No underlies the basal domain of the NT. Reports in the literature stand in agreement with a basal presentation of both Shh [202] and also BMP/activin [203,204]. In such a case, how is Shh transported through epithelial cells from the basal towards the apical domain where cilia are located and signal transduction takes place? Alternatively, are there cilia-independent modes of ligand activity perhaps taking place at the basal domain of epithelial cells?

Finally, the data discussed in this review exemplify the coupling between somitogenesis and neurogenesis at multiple stages. Future research should address the implications of these early interactions to later morphogenesis and function of the neuromuscular and neuroskeletal systems.
